# Increased neuronal nitric oxide synthase activity in retinal neurons in early diabetic retinopathy

**Published:** 2009-11-09

**Authors:** Thomas J. Giove, Monika M. Deshpande, Christine S. Gagen, William D. Eldred

**Affiliations:** Laboratory of Visual Neurobiology, Department of Biology, Boston University, Boston, MA

## Abstract

**Purpose:**

There are increased levels of nitric oxide (NO) in diabetic retinas. The purpose of this study was to determine the extent that neuronal nitric oxide synthase (nNOS) contributes to the increased levels of retinal NO in early diabetic retinopathy by examining the expression and activity of nNOS in retinal neurons after 5 weeks of diabetes.

**Methods:**

Changes in NO levels were measured using NO imaging of retinal neurons in mice with streptozotocin-induced diabetes for five weeks. NO imaging was compared to nNOS localization using immunocytochemistry, and nNOS message and protein levels were measured using quantitative real-time PCR and western blots.

**Results:**

There was a close anatomic correlation between the localization of the increased NO production and the nNOS immunoreactivity in the retinal plexiform layers of diabetic retinas. There was no change in nNOS message, but nNOS protein was decreased and its subcellular localization was altered. Treatment with insulin or aminoguanidine partially ameliorated the increase in NO in diabetic retinas.

**Conclusions:**

These results suggest that increased nNOS activity is responsible for the majority of increased NO in retinal neurons in early diabetic retinopathy. This supports a role for increased nNOS activity in the early neuronal dysfunction in the diabetic retina.

## Introduction

Diabetic retinopathy (DR) is one of the leading causes of blindness among working age adults in the developed world [1]. DR is characterized by both neuronal dysfunction and the breakdown of the retinal vasculature [2]. Complications in the vasculature are important for disease progression and are easy to detect clinically. As a consequence, many studies have approached DR as being primarily vascular in its etiology. However, retinal neuron dysfunction occurs early in DR and may even precede vascular breakdown [3,4].

Evidence of early neuronal dysfunction is demonstrated as aberrant electroretinogram (ERG) responses before any visible vascular damage in diabetic rats [4,5] and humans [6,7]. Shirao and Kawasaki [8] concluded that the oscillatory potentials (OP) were the first ERG components affected in diabetes. OP changes are a better predictor of DR in humans than fundus photography or fluorescein angiograms [9]. Evidence for changes in visual processing has been seen in as little as two weeks in diabetic rats [10], while discernable vascular changes have been noted to occur after six months to one year [11]. Similarly, there is a decrease in color and contrast sensitivity and aberrant ERGs in diabetic humans after two years [12-14], while major vascular changes do not typically occur until 5–10 years after the onset of diabetes [3].

Several neurochemical changes have also been documented early in the diabetic retina. For example, Leith et al. [15] found increased glial fibrillary acidic protein (GFAP) in Müller cells along with increased levels of glutamate and an impaired breakdown of glutamate to glutamine. There is an increase in retinal neuron apoptosis early in DR, which also precedes the vascular damage in both rodents and humans [16,17]. The presynaptic proteins synaptophysin, synapsin 1, VAMP2, SNAP25, and PSD95 all show decreases after only one month of diabetes, especially when synaptosomal fractions are selectively examined [18]. A study by Kern et al. [19] showed that early vascular damage was prevented in rats by administering the COX inhibitor, nepafenac, but retinal ganglion cell apoptosis still occurred, denoting a separation between vascular and neuronal damage.

Nitric oxide (NO) is an important signaling molecule in the vertebrate retina found to either be produced by or have effects in every retinal cell type [20]. There is evidence for increased NO in both the vitreous and aqueous humors of patients with DR [21,22]. We have previously shown that neuronal nitric oxide synthase (nNOS) is the primary source of neuronal NO and the most abundant form of nitric oxide synthase (NOS) in retina [23-25]. However, there is little characterization of nNOS in DR, and the results have been inconsistent [26,27].

In this study, we examined changes in NO and nNOS in the retinas of mice that had streptozotocin-induced diabetes (STZID) for five weeks. We found no change in nNOS message levels, but nNOS protein levels decreased and its subcellular localization was altered in the inner plexiform layer (IPL). Despite this, total NO levels quantified using the NO-sensitive dye diamino-5-methylamino-2',7’-difluorofluorescein diacetate-FM (DAF-FM) increased significantly. The increased NO production was closely anatomically correlated with the immunocytochemical localization of nNOS. The results of this study suggest that nNOS enzyme activity is upregulated early in DR and is concomitant with neuronal dysfunction.

## Methods

Unless specified otherwise, all reagents were purchased from Sigma (St. Louis, MO) or Fisher Scientific (Waltham, MA). All of the experiments were done in normal room light, with the exception of the NO imaging, which, for technical reasons, was done using dark-adapted retinas. All statistical evaluations were performed using OriginPro 8 software (OriginLab, North Hampton, MA).

### Animals

Adult (between 3 and 5 months of age), male C57BL/6 mice were purchased from Charles River Laboratories (Wilmington, MA) and were kept on a standard 12 h:12 h light-dark cycle with free access to food and water. All animals were treated using protocols approved by the Boston University Charles River Campus Institutional Animal Care and Use Committee. Mice were age matched and made diabetic by an intraperitoneal (IP) injection of 75 mg/kg streptozotocin (STZ; Alexis Biochemicals, San Diego, CA) in pH 4.5 citrate buffer; each injection was given after an 8-h fast, one injection per day for three consecutive days. Control animals received three IP injections of citrate buffer alone after an 8 h fast on the same days as the experimental animals. The animals were allowed to recover for two days before their blood glucose levels were tested with a FreeStyle Flash^TM^ blood glucose meter (Therasense, Almeda, CA). Mice were considered diabetic if their fasting blood glucose level was at or over 250 mg/dl. Animals that did not become diabetic were not used for this study. Treatment with insulin, aminoguanidine (AG), or N_ω_-nitro-L-arginine methyl ester (L-NAME) was given to subsets of animals starting one week after induction of diabetes. Thus, these treatments were given for a duration of 4 weeks up to the time of sacrifice as follows. Once a day, 2 U/kg insulin from bovine pancreas (in sterile saline; Sigma I5500) was administered via IP injection. Also once a day, NOS inhibitor L-NAME (37.5 mg/kg in sterile 0.1 M pH 7.4 phosphate buffer, PB) was administered by IP. Every day, 1 g/l AG, an inhibitor of both NOS and advanced glycation end product (AGE) formation, was administered to animals in their drinking water, which was changed daily. Animal weight and fasting blood glucose levels were recorded at the beginning and end of each experimental cycle. Final blood glucose levels and bodyweights were compared between each group using ANOVA. Post hoc analysis was performed using Tukey’s test. A p<0.05 was considered significant. Animals were euthanized in each experiment by exposing them to IsoFlow™ Isoflurane gas (Abbott Laboratories) until they were deeply anesthetized, and then they were immediately decapitated.

### Protein extraction and western blotting

Following sacrifice, the eyes were rapidly removed and placed into balanced salt solution (BSS; 137 mM NaCl, 5 mM KCl, 2 mM CaCl_2_, 15 mM D-glucose, 1 mM MgSO_4_, 1 mM Na_2_HPO_4_, and 10mM HEPES, pH 7.4) and the retinas were then surgically isolated from the eyes. Specimens in solution were immediately placed into 1.5 ml centrifuge tubes, set on dry ice. Six retinas (three animals) were pooled together to ensure an adequate amount of protein for detection by western blotting. The retinas were then suspended in 200 μl of homogenization buffer containing 20 mM Tris-HCl, 0.05% SDS, and 1X Complete Mini Protease Inhibitor Cocktail with EDTA (Roche Applied Science, Indianapolis, IN) before being ultrasonically homogenized. The protein concentration in each sample was determined using the Bradford method. In each lane, 100 μg protein was loaded onto 8% SDS PAGE gels and transferred onto 0.4 μm Immobilon™ PVDF membranes (Millipore, Billerica, MA). A rapid detection method was used, whereby the blocking step was omitted [28]. The primary antiserum was a rabbit anti-nNOS (sc648; Santa Cruz Biotechnology, Santa Cruz, CA) that was diluted 1:5,000 in 2% BSA in Tris buffered saline w/ 0.25% Tween-20 (TBST). The secondary antibody was a horseradish peroxidase-conjugated goat polyclonal antiserum raised against rabbit IgG (Molecular Probes, Invitrogen, Carlsbad, CA) at a dilution of 1:100,000. The blots were then treated with Immobilon™ HRP substrate (Millipore) and exposed to blue X-ray film (F-BX57; Phenix, Asheville, NC). To confirm equal lane loading, we stripped the blots and reprobed them with mouse anti-β-tubulin. The β-tubulin monoclonal antibody used was developed by Dr. Willi Hafler and was obtained from the Developmental Studies Hybridoma Bank. This antibody was developed under the auspices of the NICHD and is maintained by The University of Iowa (Department of Biology, Iowa City, IA). Quantitative analysis was performed using Image J image analysis software (Wayne Rasband, National Institute of Mental Health, Bethesda, MD). The difference in protein levels was evaluated using the paired Student’s *t*-test. A p<0.05 was considered significant.

### Immunocytochemistry

After euthanasia, the mouse eyes were rapidly enucleated and placed in ice-cold BSS. The anterior chamber, lens, and vitreous were then removed and the resultant eyecups were immediately placed into 4% paraformaldehyde in PB for 90 min at room temperature. Next, the eyecups were cryoprotected in 30% sucrose in PB, embedded and frozen in Optimal Cutting Temperature embedding media (OCT; Tissue-Tek, Miles, Inc., Elkhard, IN), and cut into 14 µm thick cross-sections using a cryostat and mounted on Superfrost /Plus^®^ slides (Fisher Scientific). The eyecups from the control and diabetic mice were embedded into the same block and cryosectioned onto the same slide to reduce variability when comparing changes in immunoreactivity. The asymmetry provided by this embedding method allowed us to clearly distinguish each of the eyecups in the resultant cryosections. Thus we could embedded one eye from each of 4 separate mice with different treatments in the same block.

Immunocytochemistry was performed on the retinal sections using standard methods as previously described [25,29] on retinal cross-sections mounted on slides. Immunochemical procedures were replicated, using at least three different animals (n=3), where each "n" was the average of three trials for a given animal compared to the other retinas on the same slide as described in the previous paragraph. The primary antiserum used was rabbit anti-nNOS (sc648; Santa Cruz Biotechnology) at a dilution of 1:1,000 overnight in a humid chamber at 4 °C. The slides were then washed 3×10 min in 0.1 M PB at room temperature and then incubated for 2 h in an Alexa488-conjugated goat anti-rabbit IgG secondary antiserum (Molecular Probes, Carlsbad, CA) at a dilution of 1:500. Incubation with the primary antiserum omitted served as a control for nonspecific secondary antiserum staining. Slides were washed in 0.1 M PB as before, coverslipped with glycerol, and the fluorescent signal was visualized using an Olympus Fluoview™ 300 confocal microscope (Olympus, Melville, NY). The excitation was done using the 488 nm laser line from an argon laser and the emission was visualized using a 510–550 nm bandpass filter. Ten to 14 optical sections were taken at 1 μm intervals, with the same number of optical sections always being captured and compared for both the control and experimental retinas. The laser intensity and all confocal settings were kept consistent within replicates.

The “z project” function of Image J image analysis software was used to obtain a single image from a collapsed confocal optical stack. The images were then converted to inverted grayscale so that the immunoreactivity would appear black. Mean intensity values were then recorded for each treatment and compared to the control retina on the same slide. These values were averaged together for each animal and compared to the average control mean using the paired Student’s *t*-test. A p<0.05 was considered significant. The relative intensity of immunoreactivity within each retinal layer was quantified using Image J to produce a horizontal line-profile average of the intensity of the immunoreactivity in the different retinal layers, minimizing the effect of local regional differences.

### RNA isolation and reverse transcription

The retinas were isolated in BSS as described in the immunocytochemistry methods section, although all reagents and materials were kept RNase-free. Total RNA was then isolated from the retinas using a standard Trizol™ reagent (Invitrogen, Carlsbad, CA) extraction. To obtain sufficient amounts of RNA to perform these reactions, we pooled three animals (six retinas) together for each extraction. The RNA was then treated with rDNase™ (Ambion, Applied Biosystems, Austin, TX), following the manufacturer’s instructions, to remove any DNA contaminants. The RNA was quantified using a Nanodrop™ spectrophotometer (ThermoFisher Scientific, Waltham, MA). RNA was then converted into cDNA using the Verso™ cDNA kit (ThermoFisher Scientific) and subsequently treated with 2 U of RNase H™ (ThermoFisher Scientific) at 37 °C for 20 min.

### Quantitative real-time PCR analysis

Quantitative real-time PCR analysis (qPCR) was performed using cDNA converted from 1 µg of retinal RNA as described in the previous section, using an ABI Prism^TM^ 7900HT Sequence Detection System (Applied Biosystems, Carlsbad, CA). We used a pre-designed TaqMan™ gene expression assay (Applied Biosystems) for nNOS (assay ID: Mm00435173_m1). Our normalizing control was an optimized 18s rRNA primer set that was kindly provided to us by Dr. Ulla Hansen (Boston University, Boston, MA), which was designed to work with SYBR Green™ (Applied Biosystems). All data was obtained in quadruplicate and analyzed using the Microsoft Excel^TM^ qGene template [30].

### NO imaging

NO imaging was performed as reported previously [24]. Since DAF-FM is a light sensitive dye, all aspects of the NO imaging experiments, beginning with the retinal eyecups, were done in the dark using infrared imaging, except for the actual final light stimulations. To prepare retinal slices, we enucleated the eyes and removed the anterior segment of the eye and lens under dim light. We placed the eyecups in chilled aerated artificial cerebrospinal fluid (ACSF, 125 mM NaCl, 26 mM NaHCO_3_, 3 mM KCl, 1.6 mM CaCl_2_, 1.5 mM MgSO_4_, 1.25 mM NaH_2_PO_4_, and 10 mM glucose) in the dark on ice for 1 h to allow the retinas to recover and fully dark adapt. The eyecups were then sectioned into 250-µm-thick slices. Loading of the NO-sensitive dye, DAF-FM (Molecular Probes), was done as previously described [31]. Briefly, slices were allowed to recover from slicing for at least 30 min in ACSF at room temperature and then incubated for 60 min in ACSF containing 10 µM of DAF-FM at 37 °C. Slices were then washed 3x 15 min with fresh, aerated ACSF at 37 °C.

Some slices were stimulated for 20 min with a light emitting diode (660 nm, 3Hz, 20 µW, 25% duty cycle), while a matched set of slices were kept in the dark for comparison. Light-stimulated and control sections were then fixed in the dark for 2 h with 4% paraformaldehyde in PB and then they were washed 4×15 min in 0.1 M PB at room temperature. Fluorescent images of the retinal slices were acquired using a Fluoview^TM^ 300 confocal microscope (Olympus Corporation) using a 40× water immersion objective and the Fluoview^TM^ 2.1 software. Image preparation and analysis was conducted using Image J software as described above.

To ensure unbiased thresholding of the images when comparing the different retinal regions or treatments, we assigned a threshold value, using a custom written Image J plugin, which determined the inflection point for the region of interest (ROI) of each collapsed confocal optical image stack. Briefly, on a plot of arbitrary fluorescent units obtained from the confocal microscope versus the number of pixels in the image, the inflection point was computed as a point on the curve at which the tangent crossed the curve. Fluorescent pixels above this threshold value were quantified for each ROI using the “ROI manager.”

The quantitative analysis of these collapsed image stacks was done using the freehand selection tool in Image J to select specific ROIs. At least 25 ROIs from each animal in each category and at least five animals in each treatment group were analyzed in this manner. The average number of fluorescent pixels of all images from one animal was considered as n=1. The final graphs depict the mean and standard deviation (SD) of at least n=5 animals to average out any minor local intensity differences. The difference between the control and experimental groups was analyzed using ANOVA. Post hoc analysis was performed using Tukey’s test. A p<0.05 was considered significant.

## Results

### General condition of the mice used in this study

Mice with STZID averaged a lower weight when compared to controls after five weeks, however this difference was not statistically significant (Table 1). The only statistically significant differences were between control and insulin (p=0.0004) and control and L-NAME (p=0.03). To our observation, the diabetic mice had slightly diminished body appearance and activity levels; their activity levels were returned to normal by administering insulin or AG. In addition to diminished bodyweight, the animals that received L-NAME appeared more emaciated and lethargic than their untreated diabetic counterparts (data not shown).

**Table 1:** Health characteristics of mice used in this study.

**Experimental group**	**Weight (g)**	**Blood glucose (mg/dl)**
Control	26±0.3	129±14
Diabetic	22±1	486±9
Insulin	17±2	264±14
L-NAME	21±1.8	454±23
AG	22±0.8	433±22

Mice with streptozotocin-induced diabetes (STZID) had lower weights when compared to controls after 5 weeks, but the difference was not statistically significant. The differences in weight between control and insulin treatment (p=0.0004) and control and Nω-nitro-L-arginine methyl ester (L-NAME; p=0.03) were significant. Blood glucose levels were significantly elevated in diabetes (p<0.0001) and this was significantly lowered by insulin, although it was still significantly higher than control (p<0.0001). L-NAME or aminoguanidine (AG) did not appear to affect blood glucose levels and were not significantly different from untreated diabetic. Values are presented±SEM.

Blood glucose levels were significantly elevated in diabetes (p<0.0001). These levels were significantly lowered by insulin, although it was still significantly higher than control (p<0.0001). L-NAME or AG did not appear to affect blood glucose levels and were not significantly different from untreated diabetic, but remained significantly different from control and insulin treated animals (p<0.0001; Table 1).

### NO increased after 5 weeks of STZID and was reduced by insulin, aminoguanidine, or L-NAME

**Figure 1:**
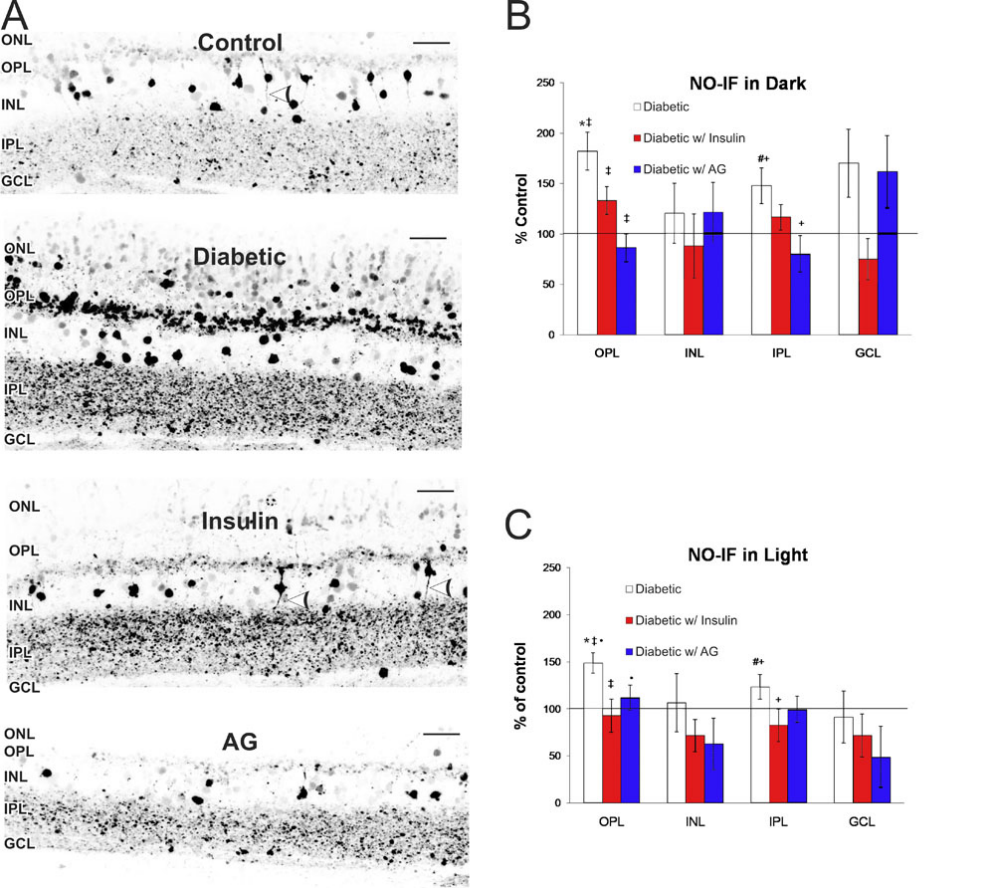
Changes in NO-induced fluorescence (NO-IF) in the diabetic retina. **A**: NO-IF increased in the diabetic retina compared to control. NO-IF in processes from somata in the inner nuclear layer (INL) were present in control (white arrowhead) but absent in the diabetic. Treatment with insulin or aminoguanidine (AG) reduced NO-IF and insulin restored NO-IF in some the processes in the INL (white arrowheads). Scale bars represent 25 μm. Quantitative analysis of NO-IF in retinal regions is shown in dark-adapted unstimulated (**B**) and light-stimulated retinas (**C**) as compared to control (100%). The outer and inner plexiform layers (OPL and IPL) depict the percent intensity, while the inner nuclear layer (INL) and ganglion cell layer (GCL) depict the percent of NO-IF labeled somata. **B, C**: Like symbols indicate statistically significant differences between groups (* represents diabetic versus control OPL, # represents diabetes versus control IPL; p<0.05). Error bars represent SD.

To examine any changes in NO levels in the retina after 5 weeks of STZID, we measured the NO-induced fluorescence (NO-IF) using the NO sensitive dye DAF-FM. We detected basal levels of NO-IF in all retinal layers in the dark-adapted retinas (Figure 1A). We observed an overall increase in NO-IF in dark-adapted diabetic retinas. In comparison to control retinas, the intensity of NO-IF in diabetic retinas increased significantly in the outer plexiform layer (OPL) by 82±19% (p<0.0001) and in the IPL by 48±18% (p=0.016; Figure 1A,B). NO-IF also appeared in the diabetic outer nuclear layer (ONL), which was usually not seen in the control ONL (Figure 1A). Interestingly, presumptive bipolar cells in the control inner nuclear layer (INL) often had NO-IF both in their somata and processes, but they appeared to have lost NO-IF in their processes in the diabetic condition. There was no significant change in the number or intensity of labeled cell bodies in the INL or ganglion cell layer (GCL) in diabetic retinas when compared to controls.

Treatment with insulin partially reduced the NO-IF in the diabetic retinas (Figure 1A,B). Insulin treatment lowered NO-IF levels in the OPL and IPL to about half that of the untreated diabetics, however this trend was only statistically significant in the OPL (p=0.01). Neither the number of cells exhibiting NO-IF nor the intensity of NO-IF in the INL and GCL were significantly different from controls. In addition, insulin restored NO-IF to some processes of somata in the INL and greatly decreased the levels of NO-IF in the ONL (Figure 1A). While insulin did help restore NO-IF to near normal levels, we did not observe a complete rescue. This was most likely because the insulin was only administered once daily. As a consequence, the blood glucose levels of the insulin treated mice were lower than the untreated diabetics, although their blood glucose levels were still elevated above 250 mg/dl (Table 1).

Treatment with L-NAME reduced retinal NO-IF to levels too low to be detected by our NO imaging method (data not shown). Treatment with AG significantly returned NO-IF to near control levels in the OPL (86±14%, p<0.0001) and in the IPL (80±18%, p=0.004; Figure 1B), but the NO-IF in the processes in the INL did not return with AG treatment as it did with insulin (Figure 1A). The number of cells exhibiting NO-IF and intensity in the INL and GCL levels were not significantly different from control.

While the results described were for dark-adapted, unstimulated retinas, we also performed the same experiments using retinas stimulated with flashing light (Figure 1C). The results were comparable to retinas in the dark, although the basal levels of NO-IF were higher in stimulated control retinas. In diabetic mice, the NO-IF in the OPL significantly increased by 49 ± 11% (p=0.0002), while in the IPL it went up by 23±13% (p=0.02). Interestingly, insulin seemed to have a stronger effect in the OPL and IPL of light-stimulated retinas, significantly lowering NO-IF to near control levels in the OPL (93±18%, p<0.0001) and in IPL (83±17%, p=0.0016). AG had a dramatic effect in the light-stimulated retinas, with intensities significantly down to 112±13% in the OPL (p=0.02) and lower (but not statistically significant) to 98±14% in the IPL (Figure 1B,C).

### nNOS protein levels decrease after 5 weeks of STZID

**Figure 2:**
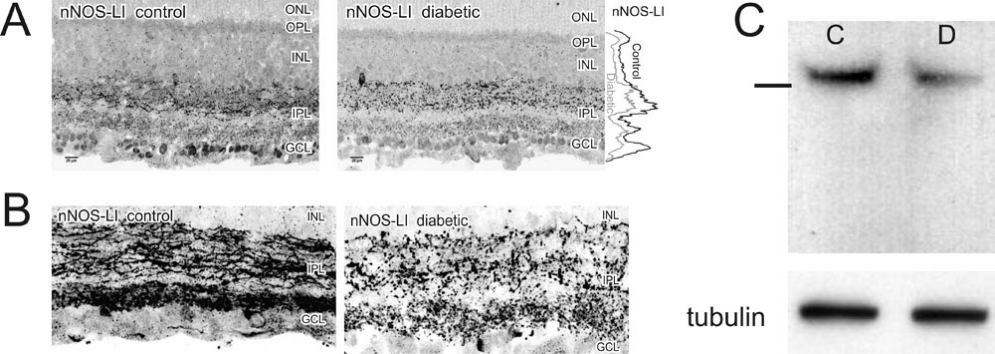
nNOS protein levels decreased after 5 weeks of diabetes. **A**: Mean fluorescence levels of nNOS immunoreactivity were significantly reduced in the outerplexiform layer (OPL), inner nuclear layer (INL), inner plexiform layer (IPL), and ganglion cell layer (GCL) when compared to controls (n=11). The graph on the right indicates the line profile average of the intensity of nNOS in each layer of the retina. **B**: nNOS immunoreactivity in the IPL no longer filled neuronal processes in the diabetic and was localized in structures which resembled synaptic boutons in size and location. **C**: western blots detected a single ~160 kDa band consistent with nNOS. Levels of β-tubulin are shown to confirm equal protein loading. There was a 24%±11 decrease in total nNOS protein (C is control, D is diabetic; n=8). Line on the left indicates the location of the 150 kDa molecular weight marker.

While NO-IF increased, nNOS-like immunoreactivity (nNOS-LI) significantly decreased by 21±11% in the OPL (p=0.005), 15±9% in the IPL (p=0.012), 19±17% in the INL (p=0.001), and 26±14% in the GCL (p=0.006; Figure 2A). These changes were not affected by treatment with insulin or AG (data not shown). There was nNOS-LI in the IPL in many apparent neuronal processes in control retinas. Interestingly, nNOS-LI was largely absent from such processes of the IPL in the diabetic condition and appeared more punctate in expression, suggesting nNOS-LI was in synaptic boutons (Figure 2B). Western blots probed with the nNOS antiserum confirmed an overall reduction of nNOS protein by 24±11% (p=0.005), consistent with our immunocytochemistry (ICC) findings (Figure 2C).

**Figure 3:**
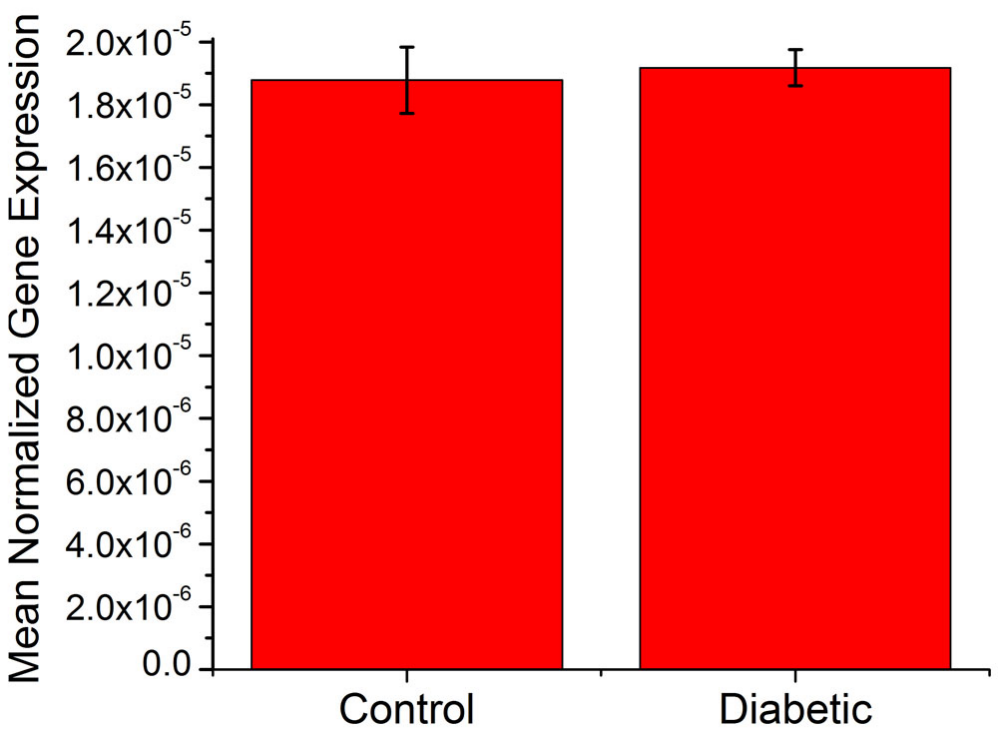
qPCR measurements of retinal nNOS transcripts using an Applied Biosystems TaqMan™ assay. No difference was seen between control and diabetic nNOS mRNA levels. The values are depicted as mean normalized expression (MNE) compared to the 18s rRNA (n=4). The data were analyzed using the qGene template [30], using the following equation:
$$\begin{array}{l} \text{MNE}=[\frac{{\left(E_{ref}\right)}^{CT_{ref}},replicate1}{{\left(E_{target}\right)}^{CT_{target}},replicate1}+\frac{{\left(E_{ref}\right)}^{CT_{ref}},replicate2}{{\left(E_{target}\right)}^{CT_{target}},replicate2}+\frac{{\left(E_{ref}\right)}^{CT_{ref}},replicate3}{{\left(E_{target}\right)}^{CT_{target}},replicate3}+\frac{{\left(E_{ref}\right)}^{CT_{ref}},replicate4}{{\left(E_{target}\right)}^{CT_{target}},replicate4}]\\ 4 \end{array}$$where; E_target_, PCR amplification efficiency of the target gene; E_ref_, PCR amplification efficiency of the reference gene; CT_target_, threshold cycle of the PCR amplification of the target gene; CT_ref_, threshold cycle of the PCR amplification of the reference gene. The CT was defined as the cycle at which the fluorescence rises appreciably above the background fluorescence and determined automatically using Applied Biosystems SDS software version 2.3.

To determine if the change in nNOS protein was due to a decrease in nNOS gene expression, we performed qPCR using an Applied Biosystem’s TaqMan™ assay. We found no difference in *nNOS* mRNA expression levels between control and diabetic retinas (Figure 3), an indication that changes in nNOS protein levels were not due to changes in its gene expression.

## Discussion

### Neuronal versus vascular complications

Several studies of DR find evidence for cellular and metabolic abnormalities in retinal neurons before vascular complications [4,8,32]. In both human and animal models of DR, retinal dysfunction can first be detected using ERGs, long before any vascular pathology becomes apparent [8]. However, permeability changes can occur as early as eight days after diabetic onset in rats [33], and future studies should consider this. Nonetheless, studies that have looked at the vasculature in greater detail did not find major vascular complications until 6 months to 21 months after the onset of diabetes in rodents [11,32,34,35].

### Role of nNOS in diabetic retinopathy

We found a large increase in NO production, which mirrored the anatomic localization of nNOS-LI, despite an overall decrease in nNOS protein levels. There have been relatively few studies examining nNOS in retinal neurons of diabetic animals, and the results have varied. One study used a combination of western blots and ICC to conclude that the number of nNOS-positive bipolar cells increases in the INL of the diabetic rat after nine weeks [26]. More consistent with our findings, an earlier study using a combination of NADPH diaphorase histochemistry and ICC found that the total number of nNOS expressing neurons decreases, and this loss is prevented by AG [27].

### nNOS is responsible for the increases in NO-IF in the OPL and IPL

It is theoretically possible that some of the NO-IF we observed was produced from inducible NOS (iNOS) or endothelial NOS (eNOS). Several studies have examined iNOS as a potential mediator of DR. However, these studies all indicated that iNOS plays a critical role in subsequent vascular damage and do not link iNOS with early neuronal dysfunction [36-38]. Du et al. [39], showed that high glucose caused an increase in iNOS in the Müller cell culture line rMC-1, but not in bovine retinal endothelial cells. Abu El-Asrar et al. [38] detect iNOS in Müller cells of human patients with DR, but not in any cells of the retinas of normal individuals. Therefore, although iNOS is clearly involved in the pathology of the diabetic retina, it is more closely associated with glial and vascular complications. To our knowledge, there is no definitive evidence linking iNOS to the early neuronal pathology seen in DR, and there has been no selective localization of iNOS within the IPL or OPL. In contrast, our NO imaging results from this and previous studies [24,25] clearly indicated that the NO-IF in retina is closely anatomically correlated with nNOS-LI—observations that support our belief that the increased NO was coming from nNOS and not iNOS.

Although we detected relative low levels of *eNOS* mRNA compared to *nNOS* mRNA in retina using qPCR and found eNOS-LI in retinal vasculature (data not shown), we did not detect eNOS in retinal neurons using several commercially available eNOS antisera (Santa Cruz Biotechnology, Inc., sc8311, sc653, and sc654; BD Transduction Labs, 610298). However, this was not surprising since previous work shows only weak immunocytochemical staining of eNOS protein in the INL and blood vessels, suggesting that it is not abundant in the retina [36,40]. Although there is evidence that eNOS is involved in vascular complications of DR, the vasculature only makes up about 3%–5% of the retina [36,41]. Most important, the increased NO-IF we observed in the plexiform layers is not likely to be produced by eNOS, because the NO-IF is clearly not associated with blood vessels.

If NO is contributing to various stages of DR, then it is extremely important to find usable inhibitors of NOS as potential therapeutic targets. Gross inhibition of NOS is not a realistic option, as we found that L-NAME almost completely eliminated NO-IF and this treatment was not well tolerated. In the present study, AG was well tolerated, and it was apparent that AG was able to inhibit nNOS. Other studies also show that AG is not exclusively an iNOS inhibitor, as it is often reported to be [42,43]. However, since AG is also an inhibitor of AGE formation [44,45], we cannot discount the role AGE formation may play in regulating NO levels.

### Potential mechanisms for increased nNOS activity

In both diabetic human [46] and rat retinas [47], there is increased NMDA receptor NR1 subunit immunoreactivity. These NMDA receptor channels provide an important route for Ca^2+^ entry, and subsequent activation of Ca^2+^-dependent intracellular enzymes such as nNOS. Neurotoxicity associated with excitatory amino acids is reported to be mediated to a large extent through the activation of NMDA receptors [48]. Since nNOS activity is Ca^2+^-dependent and calmodulin-dependent, it would be also be activated by these Ca^2+^ increases [49]. Furthermore, a subset of nNOS resides at the post-synaptic density by virtue of its PDZ-domain; this may explain why the nNOS we find remaining in the diabetic IPL appears largely synaptic [50]. Any nNOS at the post-synaptic density would be in close proximity to the largest influxes of calcium. Thus the reduction of nNOS protein may be compensated for by large glutamate-induced influxes of calcium at synapses.

Another significant possibility is that there is a post-translational activation of nNOS. nNOS enzyme activity is strongly inhibited by a Ca^2+^-dependent and calmodulin-dependent protein kinase II that phosphorylates nNOS at Ser^847^ [51,52]. Increased levels of Ca^2+^ can activate calcineurin (PP2B) to dephosphorylate nNOS at Ser^847^, which in turn allows nNOS to increase NO production [53]. Thus it is possible that increased Ca^2+^ levels can stimulate NO production in two distinct ways: by increasing intracellular Ca^2+^ to directly activate nNOS, and by activating calcineurin to activate nNOS through dephosphorylation of an inhibitory site.

### Consequences of increased NO production in diabetes

There are several potential molecular and cellular consequences of an increase in NO in synapses. One consequence of diabetes is hypoxia in the diabetic retina [54]; NO from nNOS has been shown to sensitize neurons to hypoxia-induced death via competitive inhibition of cytochrome oxidase [55]. Thus, the increased NO may contribute to the early neuronal cell death in DR. NO has also been shown to increase release of neurotransmitters through both cGMP-dependent mechanisms [56] and through Ca^2+^ independent mechanisms involving synaptic vesicle docking and fusion reactions [57]. In particular, NO has been shown to increase release of glutamate in chick retina [58]. In contrast, NO has been shown to decrease glycine release in retina [59]. Such increased levels of retinal glutamate and decreased release of glycine may also be related to the increased cell death in DR. These neurochemical changes are consistent with studies using a combination of ICC, western blots, and qPCR that report that the presynaptic proteins synaptophysin, synapsin 1, VAMP2, SNAP25, and PSD95 all showed decreases after only one month of diabetes, especially when synaptosomal fractions were selectively examined [18].

Future studies will need to determine the exact mechanisms that led to the increase in nNOS activity. In addition, it will be important to separate the timing and relative contributions of nNOS versus iNOS to get an accurate picture of how each pool of NO affects the retina as a whole. However, the results of our study indicate that nNOS may play a major role in providing excess NO in early DR. Future studies should address how this pathologically increased NO can be selectively reduced without detrimentally affecting the many normal functions of NO in the retina. This approach could provide a significant new strategy for treating the early neuronal cell loss in DR.
